# The effects of individually designed insoles on pes planus treatment

**DOI:** 10.1038/s41598-020-76767-y

**Published:** 2020-11-12

**Authors:** Mahmut Açak

**Affiliations:** Department of Coach Education, Faculty of Sport Sciences, Inönü University, Malatya, Turkey

**Keywords:** Experimental models of disease, Medical research

## Abstract

The aim of this study was to examine the effects of individually designed insole in pes planus treatment. Designed insoles was adjusted according to height, length and function of the sole of each participant with pes planus in order to improve the physical parameters of them. A total of 34 participants (17 males and 17 females) with pes planus participated in the study. Height, weight, percent body fat, 30-m sprint test, vertical jump, 12-min Cooper test and Visual Analog Scale (VAS) measurement were obtained before the study and after 1 year later. Wilcoxon signed rank test was conducted to examine whether there were any differences between the pre- and post-test measurements. It was determined that individually designed insoles reduced body weight and BMI, made positive improvements in 30-m speed, vertical jump and 12-min Cooper scores, and significant decrease in VAS scores. In conclusion, it is seen that individually designed insoles have beneficial role in normalizing forces acting on the foot and improve the physical performance parameters of individuals with pes planus. Future studies are needed to explore the long-term effects of individually designed insoles and prefabricated insoles.

## Introduction

The foot, which is one of the most complex organs of our body not only supporting body weight with 33 joints, 26 bones and more than 100 muscles but they also balance the body weight^1^. There three aches in the foot as arcus longitudinalis pars medialis, arcus longitudinalis pars lateralis and arcus transversalis^2^. As weight increases, these arches spread and collapse, recovering when the weight decreases^3^. Foot arches serve to support body weight and to push the body forward during movements as a shock absorber^4,5^. Flexible arches ensure that the foot adjusts to weight transfer and the surface pressed^4^. When the structure of the arch is deteriorated, it cannot be possible to transfer the body weight to the anterior part of the foot during walking, allow the ankle to fall toward the midline of the body and increase stress on the knees, hips, and lower back^6^.

There are three main factors that protect foot arches. These are the shape of the arch, strong bonds and muscle tone^7^. Although the elements that support the arch do not move, the suitability of the joint surfaces of connected bones helps prevent the collapse of the arch. Furthermore, the support depends on plantar ligaments and leg muscles. Therefore, it is of utmost significance to have strong leg muscles. The arch structure of the foot can be deteriorated by birth or after birth. Pes planus in adults can occur due to standing on rough surfaces for a long time as part of one’s job, choosing unsuitable shoes and in situations where plantar fasciitis is overloaded as a result of excessive weight, systemic diseases, neurological diseases and muscle imbalances^8^. It was stated that problems occurring due to pes planus affect individuals’ lives and their competencies in activities negatively^7^. Moreover, these changes can cause pain in the foot, calf and back as well as gait disorders, affecting daily activities such as exercising, standing for a long time and walking, physical fitness and quality of life negatively^9–12^. In order to overcome these negative effects of pes planus, foot orthotics are often applied inside the shoes. However, there is little agreement on the specific effect of orthotics on foot kinematics. It is thought that they can ensure healthy foot mechanics by supporting the foot and distribute the body weight equally, make it possible to perform daily activities and sports without pain and also help lower the risk of injury^13^. Because, they can control excessive or prolonged foot pronation during the stance phase of gait, minimize overstress on soft tissues and alleviate associated symptoms^14^. Moreover, it is believed that orthotic insoles influence the lower extremity movement pattern through a combination of mechanical control and afferent feedback mechanism from cutaneous receptors^15^. However, the effect of orthoses on foot movement is not yet clearly understood**.** Because arch height of everyone’s feet is different but arch height of prefabricated or custom-made insoles is standard. The use of these insoles is thought to cause different results in pes planus studies. Therefore, the unique feature of this study is that the specially designed insole can be adjusted for everyone's feet and it is believed that this specially designed insole will make a difference in pes planus treatment.

The prevalence of pes planus reported to be approximately 5 to 14% by different researchers^16,17^. Since individuals with pes planus have a passive lifestyle, they can face health problems such as cardiovascular diseases, obesity, diabetes, etc. It is a fact that such health problems cause important problems in the economy and health system of the countries. Therefore, conservative treatments such as use of insoles and orthoses, physiotherapy applications and finally surgical interventions are preferred to decrease pain and improve quality of life of individuals with pes planus^18^. However, no protocol regarding the frequency, intensity, type and duration of the exercise used in the treatment of pes planus was found. At the same time, there were limited study examining the relationship between the ground and the pes planus^19^. The objective of this study was to examine the effects of individually designed insole in pes planus treatment. It was hypothesized that individually designed insoles would improve the physical performance parameters and minimize the complaints of the individuals with pes planus.

## Methods

### Selection of the participants

A total of 48 participants were randomly selected among the individuals who applied to the Orthopedics and Traumatology Department of Turgut Ozal Medical Center in Inonu University with pes planus complaint. All participants had undergone a clinical examination by a physician. 14 participants who had history of any diseases, musculoskeletal pathologies, open wound in foot, concurrent use of in-shoe orthotics or a previous spinal/lower limb surgery were excluded from the study. Finally, 34 participants (17 males and 17 females) aged between 18 and 28 were included in the study. 10 of the participants were rigid and 24 of them were pes planus.

Ethics committee report (protocol number: 2016/138) was received from the Inonu University clinical research ethics committee to eliminate ethical concerns before starting the study. Participants were verbally informed about the aim and the implementations of the study and those who wanted to participate in the study voluntarily signed the informed consent form. The study was conducted in accordance with the Declaration of Helsinki.

### Insole design and placement

**Figure 1:**
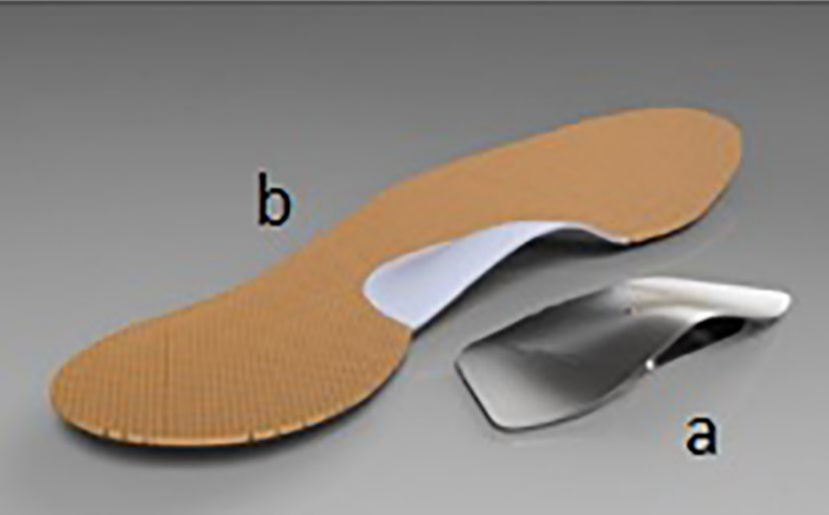
Individually designed insole. (**a**) 1 mm thick stainless chrome steel, (**b**) 3 mm thick antibacterial leather.

**Figure 2:**
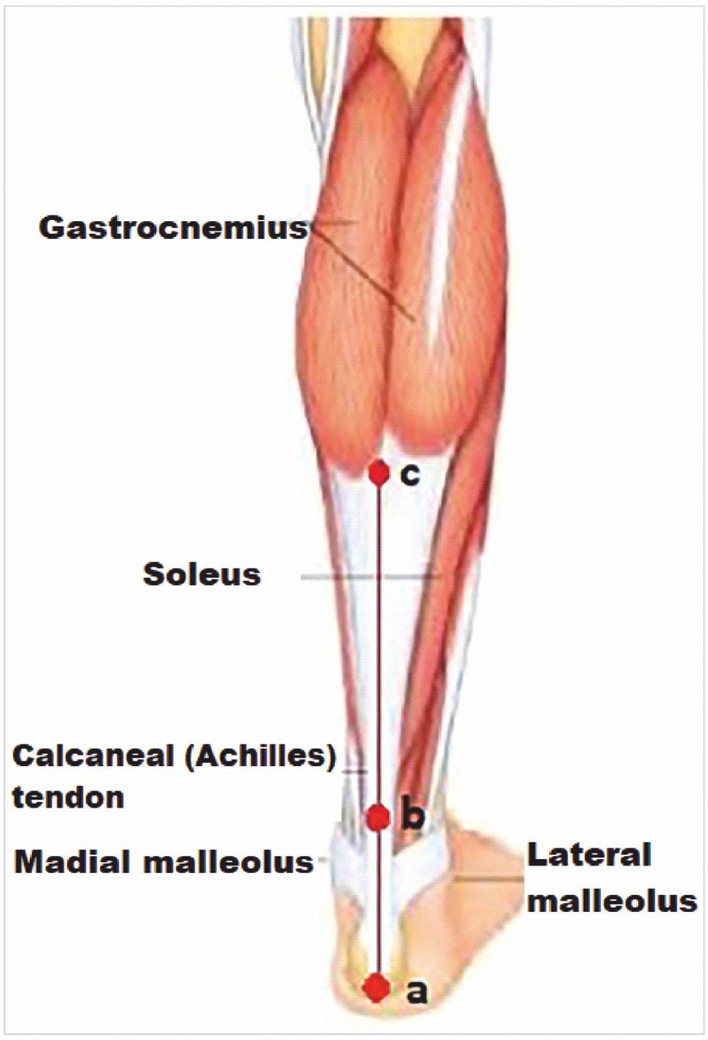
Drawing the alignment line in supine position. (**a**) mid-point of the calcaneus, (**b**) mid-point of ankle joint and Achilles tendon, and (**c**) mid-point of gastrocnemius is marked and a line is drawn with a ruler.

**Figure 3:**
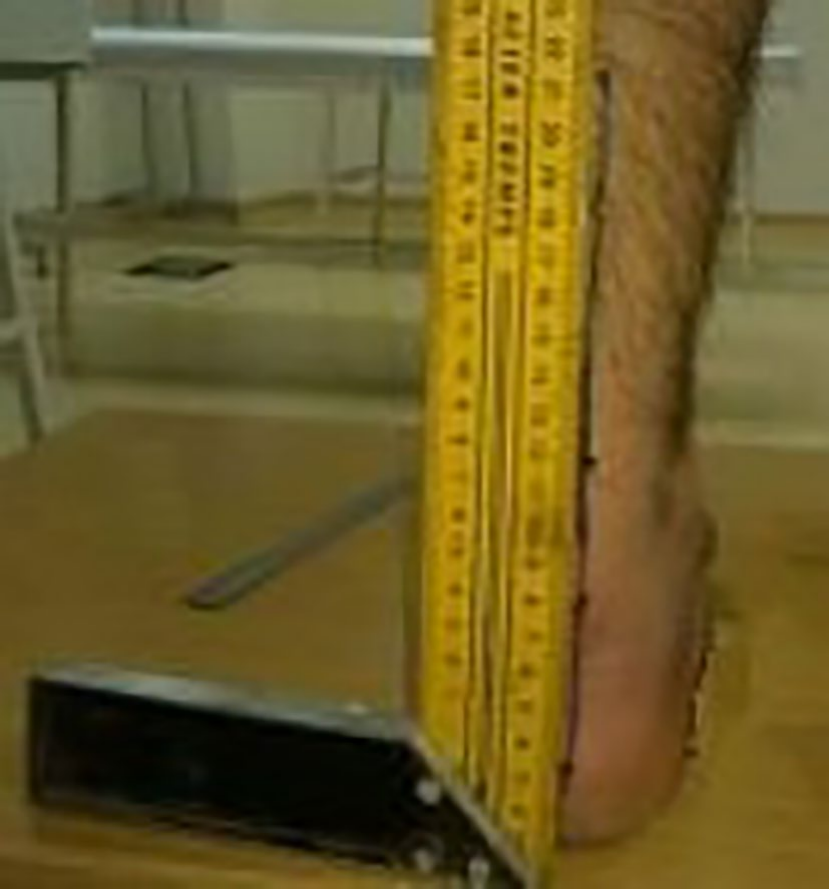
Adjustment of the height of inserted insole into the arch of foot. The height of the insole was adjusted manually until degrees of the line was 90° perpendicular to the ground, which is measured by using a miter.

Insoles are made up of 1 mm thick stainless chrome steel covered with 3 mm thick antibacterial leather to prevent damage to the foot. At the design stage, the foot structures of 130 healthy individuals were examined and a final shape of the insole were given in the pilot study (Fig. 1). Before placing the insole under the feet, middle points of the calcaneus, ankle joint and Achilles tendon is marked and a line is drawn with a ruler to the back of the leg aligned from the marked points to the gastrocnemius in a prone position (Fig. 2). Then, the participants stand up and the foot arch is raised by insole until this line is 90° perpendicular to the ground, which is measured by using a miter (Fig. 3). When 90° is achieved, the insole is fixed and placed into the shoe. Arch heights of the participants were measured individually and the designed insoles were separately adjusted for each individual. All participants were examined by a single experienced physician who worked on pes planus for years from the physical therapy and rehabilitation department.

### Measurement

Image of the soles of feet were obtained by using the podoscope device (Chinesport S.p.a., Udine, Italy) which is reliable, easy to use and increased visibility of the foot^20^. All measurement conditions such as the time of the day, hardness of the surface stood on barefoot and the temperature of the testing platform were set identical for all participants. The soles of the participants were thoroughly cleaned with alcohol before the measurement. The participants were asked to distribute their body weights equally on both feet while standing still on 5 s the glass surface and looking straight forward according to the Frankfurt plane. Then the image of the foot is recorded on the computer and the obtained image of the sole was analyzed using the Global Postural System/PODATA software with supreme precision to evaluate the overall structure of the foot^21^.

A highly reliable Staheli index (SI) was used to evaluate whether the structure of the foot was flexible, rigid or pes planus from the images obtained. The SI is the ratio obtained by dividing the narrowest width of the central foot to the widest width of the heel. The range between 0.50 and 0.70 is considered normal, whereas a ratio of over 0.70 is accepted as pes planus^22^. In order to determine the effects of the individually designed insole on the participants, height, body weight, percent body fat, 30-m sprint, vertical jump, 12-min Cooper run and pain intensity measurements of the participants were recorded both at the beginning of the study and 1 year later. The participants used the insoles every day during 1 year. When they went out, they put it in their shoes and when they came home, they put it in their slippers without any exception.

Heights of the participants were measured using a wall-mounted stadiometer (Seca, Germany) to the nearest 0.1 cm, weight of the participants were measured using electronic scale (Seca, Germany) to the nearest 0.1 kg.

Percent body fat was estimated by body mass index (BMI) which is a value obtained by dividing the participants' body weight (kg) by the square of the height (m^2^) (BMI = kg/m^2^). Finally, the calculated BMIs were evaluated according to WHO reference values.

Vertical jump test was used to determine anaerobic power of the participants. Participants instructed to jump vertically from a knee flexion of 90° and execute a maximum vertical jump while swinging the arms actively^23^. After 2–3 practice trials, 3 trials were carried out and the best trial was used for analysis.

30 m sprint test was performed outdoors in order to determine anaerobic power of the participants with Smartspeed timing system (Fusion sport, USA) between two gates placed at 30 m distance apart. Participants performed three 30 m sprint runs as fast as they could with at least three minutes rest between trials^24^. Time was recorded automatically by the system. The fastest of the three trials was used as the criterion measure.

Cooper test is one of the most widely used field tests to determine the maximum aerobic capacity (VO_2_max), and participants were instructed to cover the longest possible distance in 12 min, running preferably, but walking whenever necessary to prevent becoming excessively exhausted^25^. The test was performed outdoors. The prediction of VO_2_max was obtained using the formula VO_2_max = – 10.25 + (0.022 × distance in meters)^26^.

Visual Analog Scale (VAS) was used to measure the amount of pain that a patient feels ranges across a continuum from none to an extreme amount of pain^27^. The decrease in the score obtained from the scale indicates that the severity of pain increases.

### Statistical analysis

Data analysis was performed using IBM SPSS Statistics for Windows (SPSS, version 21.0, Armonk, NY: IBM Corp.). According to the results of Shapiro–Wilk normality test, Wilcoxon signed rank test was used to test whether there were significant differences between the pre- and post-measurements. The level of significance was accepted as p < 0.05 and results were expressed as mean ± standard deviation (x̄ ± sd).

## Results

A total of 34 participants enrolled in the study.10 of the participants were rigid and 24 of them were flexible pes planus. Mean age of the participants was 21.88 ± 2.83, height was 166.97 ± 6.45, weight was 79.65 ± 7.62 and BMI was 28.51 ± 1.19 (Table 1).

**Table 1:** Descriptive characteristics of the participants.

Parameter	n	${\overline{\text{x}}}$ ± sd	Min–max
Age (year)	34	21.88 ± 2.83	18–28
Height (cm)	34	166.97 ± 6.45	157–180
Weight (kg)	34	79.65 ± 7.62	69–99
BMI (kg/m^2^)	34	28.51 ± 1.19	25.99–31.05

Table 2 showed that there were statistically significant differences found in pre- and post-test results in weight, BMI, 30-m run, vertical jump, 12-min Cooper run and VAS measurements both in males and females according to Wilcoxon signed rank test.

**Table 2:** Pre- and post-test analysis of the participants according to Wilcoxon signed rank test.

Variable	Test	n	Males				Females			
			${\overline{\text{x}}}$ ± sd	Min–max	Z	p	${\overline{\text{x}}}$ ± sd	Min–max	Z	p
Height (cm)	Pre	17	171.88 ± 4.44	164–180	0.000	1.000	162.06 ± 3.86	157–171	0.000	1.000
	Post	17								
Weight (kg)	Pre	17	85.29 ± 6.29	78–99	− 3.637	0.000*	74.00 ± 3.52	69–80	− 3.655	0.000*
	Post	17	80.82 ± 4.97	74–90			70.18 ± 3.13	66–76		
BMI (kg/m^2^)	Pre	17	28.84 ± 1.20	26.17–31.05	− 3.621	0.000*	28.18 ± 1.11	25.99–29.74	− 3.622	0.000*
	Post	17	27.34 ± .89	25.54–29.40			26.72 ± 1.01	24.68–28.26		
30-m run (s)	Pre	17	7.32 ± .78	6.05-.8.82	− 3.337	0.001*	7.37 ± 1.15	5.10–8.61	− 2.439	0.000*
	Post	17	6.62 ± .71	5.18–7.83			6.77 ± .70	5.69–7.92		
Vertical Jump (cm)	Pre	17	24.65 ± 7.03	11–33	− 2.822	0.005*	15.94 ± 4.35	10–25	− 3.627	0.015*
	Post	17	31.65 ± 6.10	17.40			33.12 ± 4.11	25–41		
12-min Cooper run (m)	Pre	17	1077.94 ± 284.55	480–1490	− 3.623	0.000*	917.06 ± 246.01	500–1500	− 3.575	0.000*
	Post	17	2007.06 ± 316.18	1400–2450			1652.94 ± 396.26	900–2500		
VAS	Pre	17	3.65 ± .86	2–5	− 3.550	0.000*	3.59 ± 1.23	2–6	− 3.688	0.000*
	Post	17	7.29 ± 1.40	3–9			7.18 ± .73	6–8		

*p < 0.05.

## Discussion

The purpose of this study was to examine the effects of individually designed insole in pes planus treatment. At the end of the study, there were statistically significant differences found in pre- and post-test scores in weight, BMI, 30-m run, vertical jump, 12-min Cooper run and VAS measurements in both males and females. Results indicated that participants in the study had lost weight as a results of individually designed insole application. It was observed that losing weight cause increase in the rate of activities, muscle force, decrease in severity of pain and improve the quality of life of participants. Although not measured, it was believed that individually designed insole application contributed to the formation of the foot arch and increase the power of the arch to push the body forwards when 12-min Cooper run scores were examined. Because, improvements in VAS scores also support improvements in physical performance measures such as running and vertical jumping. The present findings are consistent with the results presented in Bates et al.^28^, Leung et al.^29^ and McCulloch et al.^30^, which suggested that orthoses reduce the degree of pronation in flexible pes planus. Kuhn et al.^31^ reported that custom-made flexible orthosis has significant improvements in the distribution of body weight evenly on two feet. The results of Açak and Korkmaz^32^ support the findings of study^17^. Several cadaveric studies also confirmed that orthoses improved some parameters, such as arch and hindfoot alignment^33,34–36^.

On the other hand, some authors doubt the effectiveness of the use of insole in the treatment of pes planus and their findings are different from those of the current study. Kulcu, Yavuzer and Sarmer^37^ and Chen et al.^38^ performed gait analyses and reported that there was no effect of insole in pes planus. Garcia-Rodriguez et al.^39^ showed that orthopedic treatments in children with flexible flatfoot were found useless. Kitaoka et al.^36^ reported that prefabricated foot orthosis was limited in correcting hindfoot valgus malalignment in pes planus. Brown et al.^40^ found no significant effect of arch support in pes planus.

It is known that most of the individuals with pes planus complain about pain emerging especially in dynamic activities such as fast walking and running^8^. However, Kulcu et al.^37^ reported mild to moderate calf pain after long distance walking in individuals with pes planus despite the use of silicone insoles. When the VAS score of the participants in the study are analyzed, it seems that individually designed insole is successful. This can be explained as the individually designed insole lengthens the gastrocnemius muscle or Achilles tendon, thereby reducing pain in the leg muscles. Although it is known that the foot adjust itself immediately to orthoses, the long-term effects of orthotic devices are not fully understood^41^. However, it is possible that well-fitted and supported insoles may produce a change in the structure of the foot.

## Conclusions

Pes planus is a physical problem and the arch height of each individual is different. Therefore, treatment protocols for each individual should be prepared uniquely. In conclusion, it is seen that individually designed insoles have beneficial role in normalizing forces acting on the foot and improve the physical performance parameters of individuals with pes planus. Future studies are needed to explore the long-term effects of individually designed insoles and prefabricated insoles.

## Data Availability

The datasets generated during and/or analyzed during the current study are available from the corresponding author on reasonable request.
